# Orchestrating the bone microenvironment to enhance bone-vascular coupling in critical-sized bone defect self-repairing: Advances and prospects

**DOI:** 10.1016/j.isci.2026.116297

**Published:** 2026-06-16

**Authors:** Zhiheng Xia, Mingming Zhao, Jiadong Lu, Jinyu Wang, Rongquan Duan

**Affiliations:** 1 School of Stomatology, Xuzhou Medical University, No. 209, Tongshan Road, Yunlong District, Xuzhou 221004, China; 2 The Affiliated Stomatological Hospital of Xuzhou Medical University, Xuzhou 221002, Jiangsu, China; 3 Sichuan Walk Technology Co., Ltd., Luzhou 646000, China

**Keywords:** health sciences, medicine, medical specialty, orthopedics, natural sciences, biological sciences, cell biology, integrative aspects of cell biology

## Abstract

Bone remodeling is driven by the tightly coordinated actions of osteoclast-mediated resorption and osteoblast-driven formation. During the early phase of bone defect healing, resorption predominates. Pro-inflammatory cytokines promote M1 macrophage polarization, and hypoxia-inducible factor-1α (HIF-1α) directs metabolic reprogramming to meet osteoclast energy demands. Moreover, reactive oxygen species (ROS) amplify receptor activator of nuclear factor κB (RANK) – RANK ligand (RANKL) signaling to accelerate osteoclastogenesis, and the resulting acidic microenvironment facilitates hydroxyapatite (HA) dissolution and clearance of necrotic debris. As healing progresses into the reparative phase, macrophages transition toward an M2 phenotype. During this stage, HIF-1α/vascular endothelial growth factor (VEGF) signaling and antioxidant-autophagy pathways become upregulated, enhancing cellular resistance to stress. The microenvironment also shifts from acidity toward mild alkalinity, which supports osteoblast differentiation and HA deposition. This review integrates the pleiotropic actions of osteogenic lineage cells and the spatiotemporal orchestration of bone-vascular coupling. It further examines how convergent microenvironmental stressors, including hypoxia, inflammation, oxidative stress, and acid-base imbalance, inform strategies aimed at reprogramming the bone microenvironment. These approaches strengthen bone-vascular coupling and enable a robust self-repairing program of critical-sized bone defects. By targeting these convergent stress pathways, it is possible to interrupt pathological feedback loops and establish a mechanistic framework for promoting endogenous self-repair of critical-sized bone defects.

## Graphical abstract

**Figure:**
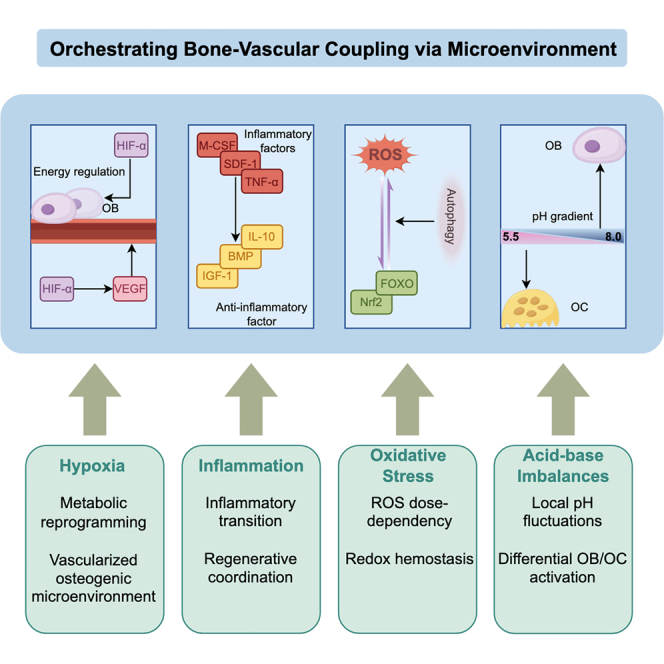

## Introduction

The bone marrow microenvironment plays a crucial role in repairing critical-sized bone defects and has garnered increasing attention. However, studies investigating its biological mechanisms often focus on single factors and lack systematic integration.

Continuous advancements in the field of bone biology and metabolism over the past few decades have progressively deepened the understanding of local microenvironment modulation to regulate the process of bone remodeling.^1^ It is feasible to surpass the critical threshold for repair and promote intrinsic autologous healing by establishing a microenvironment conducive to bone tissue regeneration.

The bone remodeling process relies critically on the coordinated activities of bone-vascular coupling and regulation of bone homeostasis. Newly formed blood vessels facilitate the transport of oxygen, nutrients, and metabolic waste. Moreover, they also aid in recruiting bone-remodeling cells for bone regeneration and bone matrix deposition. Consequently, the new bone formation consistently follows the development of capillaries.^2^^,^^3^

Osteoblasts are primarily derived from bone mesenchymal stem cells (BMSCs). They play important roles in bone homeostasis via the receptor activator of nuclear factor κB (RANK) – RANK ligand (RANKL) – osteoprotegerin (OPG) signaling pathway.^4^ Osteoblasts and osteoclasts actively drive skeletal remodeling, which involves trabecular deposition around H-type vessels and microvascular restructuring. This process also includes the infilling of gaps between resorbed and nascent bone as part of a coordinated multicellular mechanism that maintains bone homeostasis.^5^^,^^6^ Delayed union and nonunion healing in fractures are frequently attributed to inadequate vascularization.^7^ However, sometimes nonunion sites also exhibit vascularized connective tissue.^8^ This suggests that successful osteogenesis depends on the precise temporal-spatial orchestration of angiogenesis and osteogenesis.^9^

Vascular endothelial growth factor (VEGF) and basic fibroblast growth factor (bFGF) are the critical regulators of angiogenesis and can stimulate the expression of matrix metalloproteinases (MMPs) in endothelial cells. This leads to the degradation of the extracellular matrix (ECM), thereby creating space necessary for endothelial cell migration and proliferation.^3^ During osteogenesis, the H-type endothelial cells and vessels play a critical role in the regulation of coupling between angiogenesis and osteogenesis.^10^

The bone-vascular coupling maintains homeostasis in physiological conditions; however, in critical-sized bone defects, it is profoundly disrupted by a perturbed post-injury microenvironment. External factors, such as inflammation and hypoxia, can mobilize endothelial progenitor cells to promote their involvement in neovascularization. The hypoxia-inducible factor-1α (HIF-1α)/VEGF signaling pathway plays an important role in the regulatory mechanism.^8^^,^^11^ However, the synergistic crosstalk among these factors remains incompletely elucidated, particularly modulating H-type vessel formation and bone-remodeling networks. This incomplete elucidation hinders the development of targeted therapies capable of reinstating coordinated regeneration across large defects.

The regenerative outcome of critical-sized bone defects ultimately depends on the restoration of functional bone-vascular coupling within a highly perturbed post-injury microenvironment. Hao et al.^12^ classified the factors affecting the bone microenvironment into physical, chemical, and physiological factors. Among these, the four factors-hypoxia, inflammation, oxidative stress, and acid-base imbalance-collectively regulate cellular behavior and tissue homeostasis. These processes subsequently influence both vascular and osteogenic lineages. This narrative review synthesizes current knowledge on the bone microenvironment, focusing on the endogenous regulatory mechanisms underlying bone repair. Relevant literature was identified primarily through PubMed and Web of Science searches focusing on bone regeneration, osteoimmunology, angiogenesis, and microenvironmental regulation. The discussion is organized around the spatiotemporal interplay of key processes, including hypoxia, inflammation, oxidative stress, and acid-base imbalance, and examines how these factors are integrated within the bone-vascular unit. Rather than providing an exhaustive survey of the literature, this review selectively integrates studies that contribute to this conceptual framework. It aims to evaluate how these four stressors dynamically modulate bone-vascular coupling, elucidate their crosstalk, and explore microenvironment-targeted strategies to reinstate coordinated regeneration across critical-sized defects.

## Hypoxia

Hypoxia (typically defined as oxygen concentrations below 1%–2%) occurs when the local oxygen supply is inadequate to meet the metabolic demands of tissues. This leads to a failure in maintaining physiological oxygen tension and initiates a series of tissue changes and physiological responses. The HIF family affects angiogenesis and osteogenesis, thereby playing a crucial role in regulating bone remodeling under hypoxic conditions.

HIF is a sequence-specific DNA-binding protein (transcription factor) that regulates the transcription of numerous genes essential for maintaining biological homeostasis. It consists of the α and β subunits. The α subunit is sensitive to oxygen and degrades in its presence, while the β subunit, also known as the aryl hydrocarbon receptor nuclear translocator, is expressed continuously.^13^ In normoxic conditions, HIF-α degrades via the proline hypoxia hydroxylation pathway. However, the activity of prolyl hydroxylase is inhibited under hypoxic conditions. This inhibition prevents the ubiquitination and subsequent proteasomal degradation of HIF-α, resulting in its stabilization and accumulation within the cell.^14^

Recent studies have highlighted the pivotal role of HIF-1 in bone tissue regeneration. They show that metabolic reprogramming provides the energetic basis for repair, while bone cell differentiation and bone-vascular coupling serve as key mechanistic and regulatory processes, respectively.

### Metabolic reprogramming in energy supply

**Figure 1:**
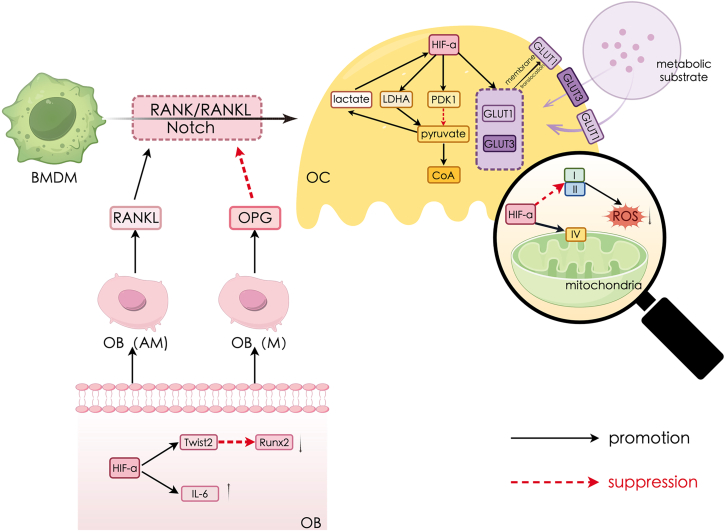
Schematic diagram of HIF-α regulating bone homeostasis (drawn by Figdraw) Abbreviations: HIF-α, hypoxia-inducible factor-alpha; Runx2, Runt-related transcription factor 2; GLUT1/3, glucose transporter 1/3; PDK1, pyruvate dehydrogenase kinase 1; LDHA, lactate dehydrogenase A; COX4, cytochrome *c* oxidase subunit 4; ROS, reactive oxygen species; OB(AM), immature osteoblast; OB(M), mature osteoblast; BMDM, bone marrow-derived macrophage; OC, osteoclast. HIF-α exerts a bidirectional regulatory effect on osteoclasts by upregulating OPG or RANKL in mature and immature osteoblasts, respectively, thereby suppressing or promoting osteoclastogenesis. Meanwhile, HIF-1α enhances glycolysis in osteoclasts by targeting GLUT1/3, PDK1, and LDHA. In addition, HIF-1α promotes mitochondrial COX4 isoform switching, thereby enhancing mitochondrial complex IV efficiency, reducing oxygen consumption and ROS production, and facilitating metabolic adaptation to hypoxia.

HIF-1α shifts the metabolism of osteogenic lineage cells and vascular endothelial cells from the citric acid cycle to glycolysis, allowing adaptation to hypoxic conditions. The mechanism involves several steps. First, HIF-1α enhances glucose uptake and accumulation by upregulating glucose transporters GLUT1 and GLUT3, thereby increasing metabolic substrate availability. It also activates pyruvate dehydrogenase kinase 1 (PDK1), which blocks the conversion of pyruvate to acetyl-coenzyme A. Simultaneously, HIF-1α induces lactate dehydrogenase A (LDHA), which promotes the conversion of pyruvate to lactate. Lactate accumulation can further enhance HIF-1α stabilization, and create a positive feedback loop that reinforces glycolysis.^15^ Second, HIF-1α regulates the mitochondrial electron transport chain (ETC) by promoting mitochondrial proteases and cytochrome *c* oxidase subunit 4 isoform 2 (COX4-2) gene expression, shifting from COX4-1 to COX4-2. This boosts complex IV efficiency. Moreover, HIF-1α reduces the activity of complexes I and II, decreasing oxygen consumption and ROS production (Figure 1).^16^

### The effect of HIF on osteogenic lineage cells

#### The effect of HIF on osteoclasts

HIF-1α has a complex association with the mononuclear-phagocyte system and osteoclasts. It enhances mitochondrial ETC activity in osteoclasts and mediates glycolytic gene transcription to fulfill cellular energy demands. Consequently, this metabolic shift facilitates adaptation to hypoxic environments.^17^ Moreover, hypoxia can boost glutamine uptake in osteoclasts.^18^ These regulatory mechanisms enable osteoclasts to generate sufficient ATP, supporting increased osteoclast numbers and promoting bone resorption. The fundamental biological significance of this HIF-1α-driven metabolic reprogramming lies in satisfying the acute bioenergetic demands of osteoclasts during the bone resorption process. The rapid, high-flux ATP generated via glycolysis supports the high energy demand of vacuolar-type proton pumps (V-ATPase), which actively extrude protons (H^+^) into the resorption lacuna. Concurrently, the lactic acid produced as a metabolic byproduct further intensifies this localized acidic microenvironment, thereby facilitating the demineralization and dissolution of the bone matrix.^19^ Furthermore, HIF-1α can facilitate the differentiation of bone marrow-derived macrophages (BMDMs) into osteoclasts by stimulating osteocytes to secrete RANKL and VEGF and through the RANKL/Notch pathway.^18^^,^^20^ Some studies suggest that HIF-1α can enhance osteoclast bone resorption, as well as induce osteoclast differentiation.^21^ Importantly, this conclusion is based on *in vitro* results. According to Chen et al.,^20^ HIF-1α could play a dual role in osteoclast regulation, exhibiting varying effects depending on osteoblast maturity; it inhibited osteoclast proliferation by stimulating OPG secretion in mature osteoblasts, while it promoted proliferation by enhancing RANKL expression in less mature osteoblasts (Figure 1). Perhaps, the discrepancy between *in vivo* and *in vitro* experiments is also the cause of the dual role. In *in vivo* experiments, the environment shows stronger buffering capacity, and the regulatory mechanisms are more complex. In contrast, *in vitro* experiments have fewer stimulus sources and simpler action mechanisms. From the perspective of metabolic reprogramming of OB, the dual role issue can be demonstrated to a certain extent. Please refer to the end of the section.

#### The effect of HIF on osteoblasts

HIF-1α′s role in osteoblast regulation remains controversial. It can indirectly promote osteoblast proliferation and differentiation by enhancing the proliferation of BMSCs. On the other hand, increased interleukin-6 (IL-6) levels in osteoblasts can also stimulate their proliferation.^22^ Xu et al.^23^ found that HIF-1α could increase Runt-related transcription factor 2 (Runx2) expression in a periodontitis environment. Studies showed that knocking out HIF-1α reduced osteoblast activity, suggesting its role in inhibiting osteoblast apoptosis.^24^ Some studies suggest that HIF-1α can negatively affect osteoblast differentiation. Its overexpression can hinder the Runx2 function by upregulating Twist2, thereby suppressing osteoblast differentiation.^25^ Overall, these complex microenvironmental effects highlight the context-dependent biphasic role of HIF-1α, shifting from a promoter to an inhibitor in bone defects. If left unbalanced, this dynamic ultimately contributes to impaired osteogenesis. Beyond its direct transcriptional regulation, HIF-1α provides a survival strategy for osteoblasts by modulating cellular energy metabolism. In severe hypoxia, HIF-1α attenuates mitochondrial respiration by reducing electron flux through complexes I and II of the ETC, thereby imposing a metabolic constraint that limits excessive ROS production. By reducing oxidative stress-induced apoptosis, HIF-1α preserves the survival of osteoprogenitor cells during the early stage of bone repair.^26^ The divergence in existing literature underscores the critical imperative for a spatiotemporal appraisal of the molecular axes and regulatory flux governing the osteogenic program. This biphasic nature corresponds to the metabolic shift between distinct healing stages. This biphasic nature corresponds to the metabolic shift between distinct healing stages. In immature osteoblasts (AM), the metabolic pattern is characterized by the relative inhibition of the ETC chain, which objectively maintains low intracellular ROS levels. This state is highly consistent with the requirements of pre-osteoblasts (Pre-OB) to maintain genomic stability and high proliferative potential. As cells transition into mature osteoblasts (M), the metabolic center shifts from expansion toward biomineralization. This shift represents a temporal reallocation of cellular resources to satisfy the functional demands of bone formation.

### The effect of HIF on endothelial cells

The blockage of upstream blood vessels or insufficient ingrowth of central vasculature in fractures and bone defects delays the delivery of oxygen and essential nutrients, establishing a persistent oxygen concentration gradient, hypoxia. Hypoxia then activates VEGF expression through HIF-1α signaling, promoting angiogenesis and vascular invasion.^27^ Beyond VEGF-mediated chemotaxis, HIF-1α-driven metabolic reprogramming of endothelial cells provides the energetic basis for vascular invasion under extreme microenvironmental stress. In the hypoxic, nutrient-deprived fracture core, endothelial cells shift from mitochondrial oxidative phosphorylation to highly active glycolysis. This metabolic adaptation not only reduces oxygen dependence but also supports differentiation into H-type vessels (CD31ˆhi Emcnˆhi), enabling endothelial cells to penetrate the fracture core and establish a vascular scaffold for subsequent osteogenesis.

H-type vessels serve as the primary mediators of osteo-vascular coupling through a high-flux metabolic profile. Within the hypoxic bone microenvironment, the stabilization of HIF-1α induces a metabolic reprogramming in endothelial cells, significantly upregulating glycolytic activity. This rapid, high-flux energy output satisfies the immediate bioenergetic demands required for intensified endothelial cell migration and proliferation, enabling H-type vessels to achieve spatial proximity to osteoprogenitor cells. Furthermore, this metabolic state sustains the paracrine secretion of essential coupling factors, such as Noggin and Delta-like 4 (Dll4), effectively integrating cellular energy metabolism with pro-osteogenic signaling pathways.^26^

In contrast, as improved local perfusion restores oxygen tension, the transition toward mature L-type vessels is governed by the prolyl hydroxylase domain (PHD)–von Hippel-Lindau (VHL) mediated degradation of HIF-1α. The suppression of HIF-1α activity downregulates its downstream effector 6-phosphofructo-2-kinase/fructose-2,6-biphosphatase 3 (PFKFB3), thereby reducing endothelial glycolytic flux and limiting proliferative angiogenic activity.^28^^,^^29^ Consequently, endothelial cells exit the proliferative state and adopt a quiescent metabolic phenotype with reduced glycolysis.

### Key regulatory factors in bone-vascular coupling

**Figure 2:**
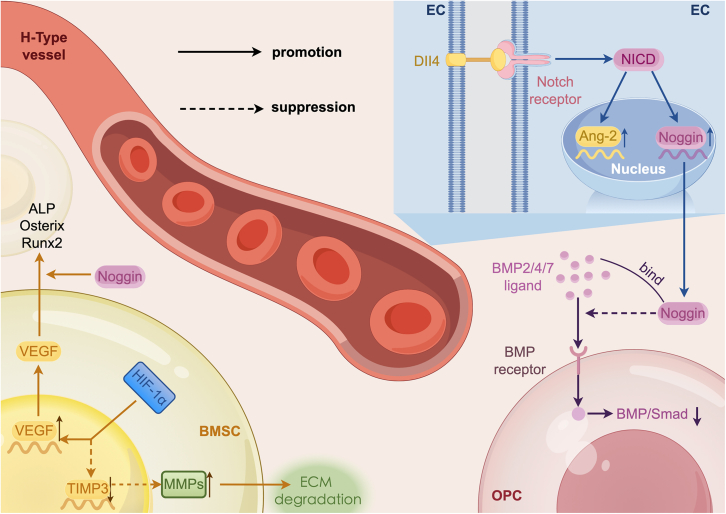
The role of HIF-α in H-type vessels formation and bone-vascular coupling (drawn by Figdraw) Abbreviations: HIF-1α, hypoxia-inducible factor-1 alpha; BMSCs, bone marrow mesenchymal stem cells; VEGF, vascular endothelial growth factor; TIMP-3, tissue inhibitor of metalloproteinases-3; MMPs, metalloproteinases; ECM, extracellular matrix. ECs, endothelial cells; Dll4, Delta-like 4; Ang-2, angiopoietin-2; BMP, bone morphogenetic protein; OPCs, osteoprogenitor cells; Runx2, Runt-related transcription factor 2. HIF-1α in BMSCs promotes H-type angiogenesis by upregulating VEGF and downregulating TIMP-3 expression. H-type ECs, activated via Dll4/Notch signaling, secrete Noggin and Ang-2. Noggin moderately antagonizes BMP/Smad signaling in OPCs, thereby promoting their proliferation and differentiation, accompanied by the upregulation of Runx2 and Osterix, as well as enhanced bone matrix deposition.^20^^,^^30^

During bone remodeling, the regulation of VEGF alone is not enough to create a stable vascular network. Stromal cell-derived factor-1 (SDF-1), another downstream target gene of HIF-1α, plays a critical role in stabilizing the link between angiogenesis and osteogenesis. In addition to regulating angiogenic factors, HIF-1α can also enhance vascularization by reprogramming the metabolism of vascular endothelial cells, thus improving their adaptability to hypoxic conditions (Figure 2).

Some studies suggest that the HIF-1α-mediated bone-vascular coupling effect might be limited to young mice.^31^ In oxidative stress-rich aged or injured microenvironments, ROS emerge as a key negative regulator of hypoxia-driven vascularization. Shao et al.^32^ demonstrated an interesting age-dependent reversal in HIF-1α′s role in bone-vascular coupling: In young bone, it could counteract the p53-mediated suppression of pro-angiogenic genes (such as VEGF), while in aged bone, it amplified p53 inhibition, thereby impairing vascularization, an effect exacerbated by ROS accumulation disrupting the HIF-1α/p53 axis. These experiments were primarily conducted at the cellular level in murine models; however, the ROS-driven mechanism also aligns with tissue and organismal level observations. These observations include age-related decline in type H vasculature, delayed fracture healing in aged models, and defective regeneration in critical-sized bone defects.^33^^,^^34^ Excessive oxidative stress can override adaptive hypoxic signaling, leading to impaired bone-vascular coupling. The reasons will be further elaborated in the section on chapter Crosstalk.

HIF-1α has a multifaceted role in bone-vascular coupling. In a localized hypoxia, HIF-1α can facilitate vascular proliferation and invasion by mediating VEGF, thereby constructing a vascular network scaffold. This alters the metabolic patterns of local cells, affecting ATP utilization and ROS formation, and affects the growth and reproduction of osteogenic cells to establish the bone-vascular coupling system. Hypoxia initiates metabolic and HIF-1α-driven adaptations essential for early vascular responses; however, inflammation subsequently dominates the orchestration of bone remodeling through immune cell polarization and cytokine signaling.

## Inflammation

After seven days of dental implant surgery, vascularized granulation tissue is evident at the defect site, accompanied by a marked upregulation of cytokines associated with immune and inflammatory responses. By day 14, woven bone formation is initiated as the granulation tissue undergoes maturation. While the expression of inflammatory and immune-related proteins persists, the cytokine profile undergoes a marked transition. It shifts from an early predominance of pro-inflammatory mediators such as tumor necrosis factor-alpha (TNF-α) and IL-6 to a later increase in anti-inflammatory factors, including transforming growth factor β (TGF-β) and interleukin-4 (IL-4) (Table 1).^35^ This suggests that inflammation persists throughout the entire bone regeneration process and may be a key driver of bone regeneration.^36^

**Table 1:** Common inflammatory factors involved in osteogenic regulation

Phase	Cytokine	Main functions	The effect on bone regeneration during inflammation	Key pathways	References
Inflammatory phase Recruitment and clearance	PDGF	chemotaxis	recruits neutrophils and initiates the inflammatory cascade	PI3K/Akt	Graham et al.^37^; Xie et al.^38^
	SDF-1	pro-inflammatory /chemotactic/transdifferentiation	orchestrates targeted homing of MSCs and serves as a critical bridge to initiate osteogenesis and coupled angiogenesis.	SDF-1/CXCR4 axis–MAPK/ERK1/2 (migration/angiogenesis);	Zhang et al.^39^; Qin et al.^40^
	IL-6	pro-inflammatory	in indirect ways and presented as elevating the RANKL/OPG ratio. high RANKL level stimulates osteoclast formation.	NF-κB/JAK-STAT	Sims^41^
	TNF-α	pro-inflammatory	high concentration: inhibits differentiation and bone formation. Promotes osteoclastogenesis and bone resorption.	high concentration: Wnt/β-catenin	Zhang et al.^42^; Qin et al.^43^
		pro-reparative	low concentration: promotes MSC osteogenic differentiation.	low concentration: EphB4/TNFR2/ERK/MAPK	
	IL-9	pro-resorptive	promotes the differentiation and resorption capacity of osteoclasts.	RANK/RANKL/OPG	Sapra et al.^44^
	M-CSF	chemotaxis	in the early stage, M-CSF orchestrates the recruitment and sustained survival of the monocyte/macrophage lineage to the injury site. However, their sustained activity can limit excessive osteoclastogenesis, maintaining the delicate balance required for the transition from resorption to formation	ERK/Akt	Starlinger et al.^45^; Zhu et al.^46^
	TGF-β	chemotaxis	rapid release: promotes early MSC recruitment, proliferation, and initial osteogenic commitment.	non-canonical: MAPK (p38/ERK/JNK), TAK1	Yoshida et al.^47^; Wan et al.^48^
Recovery period Vascular path construction	PDGF	repair promotion	recruits MSCs and Pericytes to the injury site, promotes angiogenesis, and inhibits osteoblast apoptosis.	MAPK/ERK	Graham et al.^37^; Xie et al.^38^
	IL-4 IL-13	anti-inflammatory	promotes M2 polarization of macrophages.	JAK/STAT6	Xu et al.^49^
	IL-10	anti-inflammatory	promotes osteoblast differentiation and inhibits the activity of osteoclasts.	NF-κB, JAK-STAT	Ni et al.^50^
	TGF-β	anti-inflammatory /repair promotion	sustained high levels suppress terminal mineralization, maintaining matrix permissiveness for H-type vessel invasion and bone-vascular coupling.	Canonical: Smad2/3 (ALK5); HDAC4/5/Smurf1 mediated Runx2 degradation	Wu et al.^51^
	BMP	osteogenic driver	dose-dependently promotes the proliferation and differentiation of MSCs, osteoprogenitor cells, and supports angiogenesis.	Canonical:Smad1/5/8–Runx2/Osterix	Wu et al.^51^; Garg et al.^52^
		remodeling mediator	promotes osteoclastogenesis and resorptive activity; contributes to coupled bone remodeling.	non-canonical: MAPK/p38 + RANKL/NF-κB crosstalk	
	TGF-α	promote healing	promotes epidermal healing and angiogenesis; concurrently inhibits cartilage and bone formation to prevent premature mineralization, thereby ensuring balanced callus development.	MAPK, PI3K/Akt	Singh and Coffey^53^
	IL-6	pro-inflammatory /anti-inflammatory	stimulates RANKL expression in osteoblast precursors. Physiological concentrations of IL-6 promote a balanced RANKL/OPG ratio.	Smad/STAT3	Sims^41^
Bone formation period Absorption and reconstruction Long-term steady state	IL-4 IL-13	repair promotion	suppresses osteoclastogenesis.	JAK/STAT6	Xu et al.^49^
	TGF-β	repair promotion	maintains matrix permissiveness for H-type vessel invasion and bone-vascular coupling.	non-canonical: MAPK (p38/ERK/JNK), TAK1	Wu et al.^51^
	IL-10	repair promotion	promotes osteoblast differentiation and inhibits the activity of osteoclasts.	NF-κB, JAK-STAT	Ni et al.^50^
	IGF-1	repair promotion	promotes osteogenic differentiation of MSCs, suppresses adipogenic differentiation of MSCs, enhances angiogenesis, and facilitates the conversion of cartilage into bone tissue. enhances osteoclastogenesis (but net anabolic via balanced remodeling)	PI3K/Akt–mTOR (osteogenic dominant); Wnt-catenin crosstalk	Wang et al.^54^; Ruan et al.^55^

### Inflammatory response in the bone regeneration process

#### Acute phase

The initial inflammatory phase establishes a pro-inflammatory microenvironment and eliminates bacteria and necrotic tissues, thereby triggering early infiltrates such as neutrophils, the secretion of platelet-derived growth factor (PDGF), and SDF-1 (Table 1) establishes a pivotal chemotactic gradient. This gradient does not merely recruit immune cells but specifically orchestrates the homing of mesenchymal stem cells (MSCs) and endothelial progenitors to the injury site.^56^ At this stage, M1 macrophages predominate. They activate the nuclear factor kappa-B (NF-κB) pathway by secreting pro-inflammatory cytokines, such as IL-6 and TNF-α. They function as biphasic regulators. Their high-dose surge during this acute stage is essential to drive the catabolic “clearing” of the microenvironment. This process is essential to vacate the niche for upcoming angiogenic and osteogenic activities. As this surge subsides, the transition to low-concentration TNF-α (Table 1) continues to recruit MSCs to accumulate at the injury site, thus facilitating the transition to the subsequent tissue repair phase.^57^^,^^58^

#### Recovery period

During the fracture healing process, the inflammatory response gradually diminishes. Macrophages polarize toward the M2 phenotype under the influence of cytokines, such as IL-4 and interleukin-13 (IL-13), and Janus kinase/signal transducer and activator of transcription 6 (JAK/STAT6) pathway. In the early repair phase, MSCs migrate to the injury site, become activated, and proliferate under the induction of PDGF signaling. Notably, beyond its chemotactic role, PDGF crucially inhibits osteoblast apoptosis via the mitogen-activated protein kinase (MAPK)/extracellular signal-regulated kinase (ERK) pathway to secure the regenerative cell pool. M2 macrophages promote tissue repair by secreting anti-inflammatory and regenerative factors. Among these, IL-10 and TGF-β suppress excessive inflammation and maintain matrix permissiveness. Concurrently, VEGF acts to promote local angiogenesis. BMP-2 and insulin-like growth factor-1 (IGF-1) act as the primary engines to trigger the lineage commitment of MSCs into osteoblasts (Table 1). Notably, while TGF-β is essential for early matrix assembly, its high concentration at this stage acts to temporarily inhibit terminal mineralization, thereby preventing premature bone formation and ensuring a high-quality regenerative niche (Table 1). This osteogenic process is executed via the canonical Smad1/5/8–Runx2/Osterix axis.^59^ At this stage, the low TNF-α levels in the microenvironment further enhance the osteogenic differentiation potential of MSCs by activating the p38/MAPK pathway.^42^

#### Bone formation period

During the bone formation period, new bone undergoes gradual mineralization; this process is critically regulated by osteoblasts, osteoclasts, and M2 macrophages. Osteoblasts, stimulated by BMP-2 and Wnt3a, secrete alkaline phosphatase (ALP), which facilitates the deposition of the bone matrix.^52^ RANKL, secreted by osteoblasts and MSCs, activates osteoclasts, thereby contributing to bone resorption. To prevent excessive bone loss, M2 macrophages release IL-10 to suppress osteoclastogenesis via the NF-κB pathway (Table 1). Notably, TGF-β orchestrates the coupling of terminal mineralization and vascular maturation, thereby stabilizing the nascent vasculature and ensuring long-term bone homeostasis (Table 1).^60^

### Hyperinflammation

The inflammatory acute phase of fracture healing typically lasts from a few hours to several days. Subsequently, pro-inflammatory factors gradually diminish, and the bone microenvironment transitions into the repair phase. However, inflammatory signaling pathways, such as TGF-β signaling, remain persistently activated in cases of severe tissue damage, systemic infection, or comorbid diabetes,^61^^,^^62^ impairing critical regenerative processes, including macrophage polarization toward the M2 phenotype. Due to space constraints, this section focuses on TGF-β and TNF-α as representative pro-inflammatory cytokines to illustrate their biphasic effects in bone remodeling under excessive inflammatory conditions. Other cytokines also play critical roles; however, they are beyond the scope of this study.

#### TGF-β

In inflammatory environments, the regulation of bone-vascular coupling by TGF-β exhibits a critical concentration-dependent switch. Medium and low concentrations of TGF-β enhance the expression of key transcription factors, including Runx2 and Osterix, via the TGF-β/Smad2/3 signaling pathway. This enhancement promotes the osteogenic differentiation of MSCs while maintaining a permissive niche for H-type vessel infiltration.^63^ However, when the inflammatory response is excessive, elevated concentrations of TGF-β may induce the loss of bone mass and pathological fibrosis, which fails to support bone-vascular coupling by suppressing the nuclear receptor subfamily 4 group A member 1 (NR4A1) and Wnt4/β-catenin signaling pathways. NR4A1, an endogenous inhibitor of TGF-β, normally restricts pathological fibrosis to maintain a coupling-permissive environment; however, this protective mechanism is frequently overwhelmed during chronic or excessive inflammation.^64^

#### TNF-α

During the acute inflammatory response phase, which occurs within 24 h after trauma and persists above baseline levels for up to 72 h, TNF-α plays a crucial role in recruiting MSCs and promoting angiogenesis.^65^ In the later stages, it activates osteoclasts to participate in bone remodeling. However, prolonged exposure to high TNF-α concentrations leads to the sustained activation of the NF-κB pathway, resulting in the suppression of osteogenesis-related factors, such as Runx2 and Osterix. Furthermore, TNF-α can induce the expression of Dickkopf-1 (DKK-1), a Wnt signaling antagonist, thereby inhibiting the nuclear translocation of β-catenin and blocking osteogenic differentiation.^43^ In addition, it can also over-activate osteoclasts via the RANK-RANKL-OPG pathway, resulting in uncoupled bone remodeling and impaired vascular stability.^66^

### Selective autophagy: Anti-inflammatory regulation in the human body

Distinct from macro-autophagy and micro-autophagy, chaperone-mediated autophagy (CMA) functions as a metabolic defense mechanism that is impaired under chronic TNF-α-mediated inflammatory stress.^67^ However, lysosome-associated membrane protein 2A (LAMP2A) overexpression can reverse the inhibitory effects of elevated TNF-α levels on osteogenesis. Although CMA does not directly affect the proliferation of MSCs, the genetic ablation of LAMP2A significantly downregulates the expression of key osteogenic markers, such as collagen type I alpha 1 chain (COL1A1) and Runx2. It simultaneously upregulates peroxisome proliferator-activated receptor gamma (PPARγ) expression, thereby promoting the adipogenic differentiation of MSCs. Based on this, Hang et al.^68^ found that LAMP2A overexpression could counteract TNF-α-induced hyperactivation of osteoclasts via the phosphoinositide 3-kinase (PI3K)/protein kinase B (AKT)/glycogen synthase kinase-3 beta (GSK3β) signaling pathway, protect mBMSCs from TNF-α-mediated suppression, and promote the nuclear translocation of β-catenin to enhance osteogenic differentiation. Furthermore, HIF-α is a CMA substrate. By degrading HIF-α, which accumulates under hypoxic conditions, CMA inhibits excessive bone resorption of osteoclasts and suppresses adipogenic differentiation of MSCs. In parallel with inflammation’s cytokine-mediated regulation of bone-remodeling phases, oxidative stress emerges as a downstream consequence. The resulting accumulation of ROS further modulates cellular fate and key signaling pathways.

## Oxidative stress

Oxidative stress denotes an inconsistency between oxidation and antioxidant activities, leading to cellular and molecular damage, playing a pivotal role in impaired bone-vascular coupling during aging and pathological repair.^69^ Among various reactive species, ROS emerge as the primary mediators that perturb the regenerative microenvironment by uncoupling angiogenic and osteogenic processes.^70^ Moreover, its regulation within the bone microenvironment significantly affects the remodeling process. This section examines the mechanisms by which local ROS can modulate bone remodeling in the bone microenvironment.

### Origin and nature of reactive oxygen species

ROS are primarily produced through the catalysis of redox enzymes and the mitochondrial oxidative respiratory chain.^71^ The biological impact of ROS is intrinsically dualistic and concentration-dependent. At elevated levels of ROS concentrations, mitochondrial lysis occurs, significantly reducing both its length and density, which leads to increased ROS production, impaired cellular metabolism, and potential cell death. Conversely, at physiological levels, ROS acts as a “second messenger” by regulating intracellular signaling pathways, affecting cell proliferation and differentiation.^72^ However, excessive ROS can worsen inflammation, accelerating damage to both small and large blood vessels.^73^ In response to localized increases in ROS concentration, organisms can enhance signaling pathways, such as NF-κB, MAPKs, PI3K/Akt/mammalian target of rapamycin (mTOR), and Kelch-like ECH-associated protein 1 (Keap1)-nuclear factor erythroid 2-related factor 2 (Nrf2)-antioxidant response element (ARE) signaling pathways, to strengthen cellular antioxidant defenses. They also mitigate oxidative stress by boosting the expression of phase II detoxifying enzymes and suppressing ROS production.^74^ In bone remodeling, ROS actively modulates key cellular processes. It inhibits the differentiation of BMSCs by suppressing Runx2 and ALP expression. ROS induces apoptosis in osteoblasts by activating the mitochondrial apoptotic pathway. Additionally, ROS can also promote the fusion of BMDMs into multinucleated osteoclasts by activating the MAPK/NF-κB signaling pathway; this leads to the upregulation of nuclear factor of activated T-cells cytoplasmic 1 (NFATc1), cathepsin K (CTSK), and MMP9, thereby enhancing osteoclastogenesis.^75^

### Signaling pathways involved in the regulation of bone remodeling under oxidative stress

**Figure 3:**
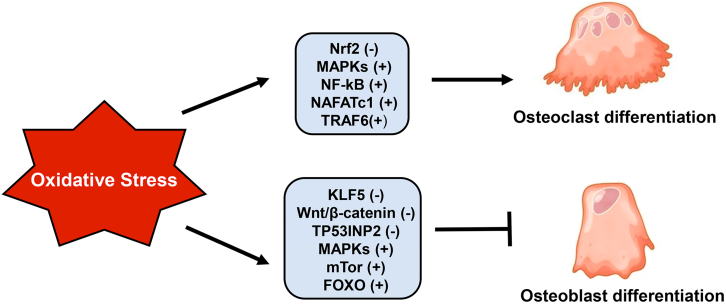
Key molecular factors mediate the effects of oxidative stress on osteoclasts and osteoblasts differentiation Adapted from Iantomasi et al.^76^ licensed under the CC BY 4.0 license. (−) represents downregulation; (+) represents upregulation. Abbreviations: FOXO, forkhead box O; KLF5, Krüppel-like factor 5; MAPKs, mitogen-activated protein kinases; mTOR, mammalian target of rapamycin; NFATc1, nuclear factor of activated T-cells cytoplasmic 1; NF-κB, nuclear factor kappa-B; Nrf2, nuclear factor erythroid 2-related factor 2; TP53INP2, tumor protein p53-induced nuclear protein 2; TRAF6, tumor necrosis factor receptor-associated factor 6.

Under oxidative stress, excessive ROS levels can perturb signaling pathways, such as Nrf2 and NF-κB signaling pathways, thereby disrupting cell proliferation, differentiation, and bone homeostasis. This modulation can act directly on the differentiation of Osteogenic lineage cells and indirectly by altering the antioxidant-autophagy axis (Figure 3).

#### Regulation of osteoclast differentiation by signaling pathways

**Figure 4:**
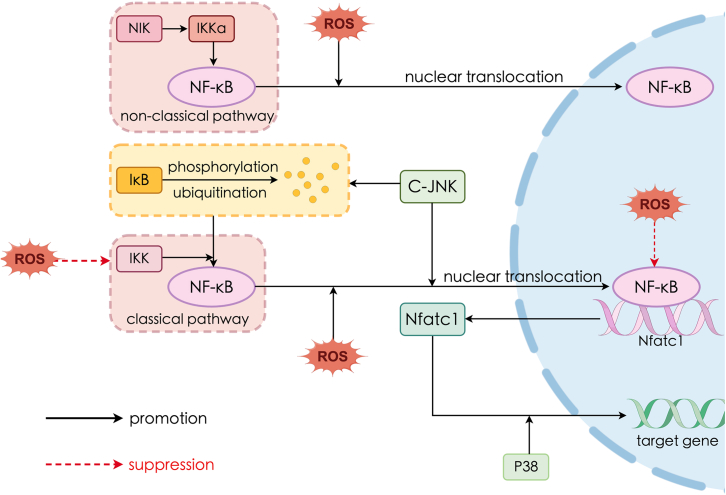
Schematic illustration of the RANK/RANKL/OPG signaling axis and its downstream pathways in osteoclasts (illustrated by Figdraw) Abbreviations: ROS, reactive oxygen species; NFATc1, nuclear factor of activated T-cells 1; IκB, inhibitor of κB; NIK, NF-κB-inducing kinase;c-JNK, c-Jun N-terminal kinase; IKK, Inhibitor of nuclear factor kappa-B kinase; IKKα, IκB kinase α. ROS exhibits spatially specific bidirectional regulation in osteoclasts. Cytoplasmic ROS promotes NF-κB nuclear translocation and NFATc1 expression through both the canonical pathway, via IκB degradation, and the non-canonical pathway, via NIK activation, thereby driving osteoclast differentiation. This process is further regulated by JNK signaling. In contrast, nuclear ROS oxidatively modifies NF-κB, thereby inhibiting its DNA-binding activity.^81^^,^^82^ Overall, ROS exerts distinct regulatory effects depending on its subcellular localization and position within the signaling cascade.^83^

Under oxidative stress, the elevated ROS in BMDMs triggers downstream signaling pathways, including NF-κB, MAPKs, and Akt.^77^ ROS facilitates regulatory changes within the NF-κB pathway by targeting essential components, such as the IκB kinase (IKK) complex, inhibitor of κB (IκB), and NFATc1, thus promoting the proliferation and differentiation of BMDM (Figure 4). Notably, ROS modulate NF-κB signaling in a concentration-dependent manner, particularly in the early inflammatory phase. Specifically, while physiological ROS stimulate the IKK complex to drive osteoclastogenesis,^78^ intense accumulation (as seen in ischemic models) can transiently inhibit IKKβ activity and IκB phosphorylation, leading to paradoxical inhibitory effects.^78^ This suggests that the early oxidative burst may function as a feedback mechanism to restrict osteoclast overactivation at the very onset of injury. Beyond the initial feedback, the MAPK pathway also plays a central role in osteoclast differentiation and is tightly connected to the NF-κB pathway described above. For instance, *c*-Jun N-terminal kinase (c-JNK) enhances phosphorylation and ubiquitination of IκB, facilitates nuclear translocation of the NF-κB dimer, and promotes NFATc1 expression. In addition, p38-mediated phosphorylation of NFATc1 induces its nuclear translocation and activates transcription of 22 downstream genes (Figure 4).^79^ In addition to enhancing the expression of genes associated with their proliferation, ROS exhibit certain additional effects on osteoclasts. Studies have shown that the accumulation of ROS can induce calcium oscillations and prompt the nuclear translocation of NFATc1.^80^ By integrating these coordinated signals, ROS at adaptive levels do not merely act as stress factors but as essential regulators that drive the initiation of the bone remodeling cycle.

#### Regulation of osteoblast differentiation by signaling pathways

At the onset of oxidative stress, osteoblasts initiate a survival program to counteract elevated ROS levels and maintain skeletal homeostasis. This adaptive response is primarily modulated by the cytoprotective pathways, including forkhead box O (Foxo) and Nrf2 signaling pathways.^76^ Specifically, the regulatory antioxidant pathways, including Nrf2 and AMP-activated protein kinase (AMPK) pathways, are involved in local bone remodeling. Within this framework, AMPK functions as an energy sensor that responds to metabolic stress and regulates antioxidant enzyme expression via Nrf2-mediated pathways. Subsequently, Nrf2 drives the transcription of phase II detoxifying enzyme genes through AREs.^84^ To further reinforce the defense, Autophagy degrades KEAP1 in the KEAP1-Nrf2 complex, thereby liberating Nrf2 to induce antioxidant enzyme genes, such as *Sqstm1*, *Sesn2*, and *Pin*k. Crucially, the functional integrity of these antioxidant defenses dictates the bifurcation of cell fate between survival and programmed death. Once ROS-induced stress exhausts the buffering capacity of the Nrf2/Foxo axis, it triggers a mechanistic switch toward NLR family pyrin domain-containing 3 (NLRP3) inflammasome assembly. This leads to the activation of Caspase-1 and the subsequent cleavage of Gasdermin D, ultimately culminating in pyroptosis.^85^ In contrast to this terminal pathway, the Foxo family serves to maintain skeletal homeostasis by enhancing free radical scavenging and upregulating DNA repair enzymes, which are essential for forestalling such irreversible damage. Ambrogini et al.^86^ showed that the mice with early knockout of Foxo1, Foxo2, and Foxo4 showed varying degrees of oxidative stress damage and increased bone resorption. In contrast, Foxo3 overexpression reduced oxidative stress and osteoblast apoptosis while increasing osteoblast numbers.

As oxidative stress persists and ROS progressively accumulate within the defective microenvironment, the role of cytoprotective mechanisms undergoes a fundamental transition. Autophagy, which is under the coordinated control of multiple signaling pathways, has a dual role in responding to oxidative stress. At low ROS concentrations, it supports cell survival by metabolizing substrates to produce energy, while it can trigger apoptotic cell death if the ROS level exceeds a critical threshold. Specifically, H_2_O_2_-induced oxidative stress suppresses the expression of the tumor-related gene tumor protein p53-induced nuclear protein 2 (TP53INP2) in BMSCs in a concentration-dependent manner (100–300 μM), thereby impairing their osteogenic differentiation potential.^87^ This inhibitory effect occurs because excessive ROS promotes the autophagy-mediated degradation of *TP53INP2* under oxidative stress, thereby inhibiting the differentiation of BMSCs into osteoblasts.^87^ The persistent autophagic degradation of regulatory proteins such as *TP53INP2* eventually dismantles the molecular scaffolds necessary for osteogenic commitment. The mechanism involves the ROS-mediated suppression of the Wnt/β-catenin pathway, which alters the expression of downstream genes, such as *OPG* and *TP53INP2,* directly impairing osteoblast function. This functional impairment of osteoblasts, coupled with the previously discussed HIF-p53 axis flip, creates a signaling void in the microenvironment. Without the necessary pro-angiogenic factors, e.g., VEGF from healthy osteoblasts, the recruitment and formation of H-type vessels are severely hindered, thereby initiating the uncoupling of the bone-vascular unit.

### The regulatory pathways of ROS on vascular endothelial cells

#### Physiological level of ROS

During the early stages of fracture healing, physiological levels of ROS function as indispensable signaling molecules for tissue repair and angiogenesis. For H-type vessels, which are the primary orchestrators of bone-vascular coupling, adaptive ROS act as crucial second messengers that enhance VEGF receptor 2 (VEGFR2) activity. The binding of VEGF to its receptor transiently induces further ROS production in endothelial cells, creating a self-amplifying positive feedback loop that effectively drives vascular sprouting. Furthermore, ROS actively participates in VEGF-induced Akt and ERK1/2 signaling pathways, promoting endothelial cell proliferation and survival. Beyond the highly active H-type vessels, physiological ROS are also critical for maintaining the integrity of the endothelial cytoskeleton and barrier function, a redox-dependent mechanism that stabilizes the low-metabolic L-type sinusoidal vessels.^88^^,^^89^

#### Excessive level of ROS

The destructive impact of excessive ROS on the bone-vascular unit is not uniform but exhibits a distinct pattern of selective vulnerability fundamentally dictated by the intrinsic metabolic states of endothelial subpopulations. H-type endothelial cells, characterized by their high proliferative activity and dense mitochondrial distribution, operate under a higher baseline of endogenous ROS production to support active bone-vascular coupling. This intense metabolic load results in a significantly narrower redox buffer compared to their L-type counterparts. Consequently, when external oxidative stress is imposed, H-type cells become the primary and most immediate targets. The additional ROS load quickly exceeds their antioxidant capacity. This overload directly triggers extensive DNA damage and lipid peroxidation.^90^ This leads to the selective apoptosis of the H-type vessels and the subsequent collapse of the pro-osteogenic microvascular network. On the other hand, L-type vessels exhibit greater tolerance to ROS-induced apoptosis due to their lower metabolic baseline. However, excessive ROS selectively target and degrade their endothelial junction proteins, leading to a significant increase in vascular permeability. This structural compromise results in extensive vascular leakage and dilation.^91^

### The regulation of bone-vascular coupling by oxidative stress

The ROS-mediated regulation of proliferation and differentiation is complex, involving the transcriptional and translational regulation of target genes and modulation of autophagy, apoptosis, and other cellular processes. Importantly, ROS exerts concentration-dependent effects on multiple signaling pathways, fundamentally reflecting a dynamic, stress-adaptive self-regulatory mechanism of cells during fracture healing. From a spatiotemporal perspective, failure to precisely regulate ROS levels within the post-fracture microenvironment disrupts bone-vascular coupling. This disruption impairs effective angiogenesis-osteogenesis coordination and ultimately prevents the formation of mechanically robust new bone.

Therefore, the accurate assessment of ROS effects on cell differentiation under oxidative stress requires careful consideration of cell type, ROS concentration, and exposure duration. In addition to ROS-driven disruptions, acid-base imbalances can also introduce pH-dependent constraints on enzymatic activity and mineralization, thus collectively perturbing the regenerative microenvironment.

## Acid-base imbalances

Under normal physiological conditions, the bone repair microenvironment exhibits distinct spatiotemporal pH heterogeneity. During the early stage, it is locally acidic (pH 5.5–6.5), which promotes osteoclast-mediated bone resorption to remove necrotic tissue and prepare for regeneration. The microenvironment gradually becomes weakly alkaline (pH 7.4–8.0) in the subsequent repair phase, thereby facilitating osteoblast differentiation and directional deposition of hydroxyapatite (HA) crystals. This acid-base homeostasis is essential for effective bone regeneration.

### Physiological acidic microenvironment (pH 5.5–6.5)

After a severe bone injury, the blood vessels in the bone and surrounding soft tissues rupture and form hematomas. Necrotic tissues recruit immune cells from the surrounding soft tissues and the lymphatic system. These recruited cells secrete various cytokines to initiate an inflammatory cascade. Consequently, a temporary hypoxic and physiologically acidic microenvironment (pH 5.5–6.5) develops during the early fracture stage.^92^

#### The effects on osteoblasts and osteoclasts

The early acidic bone microenvironment activates osteoclast-mediated bone resorption to clear necrotic tissues, including bone fragments. Concurrently, it temporarily inhibits osteogenesis-related ALP activity and matrix mineralization. This inhibition prevents the early hematoma from ossifying prematurely, establishing a permissive foundation for subsequent and orderly bone formation.

In addition to temporarily inhibiting bone formation, this microenvironment also exhibits osteoclasto genesis - osteogenesis coupling. Osteoblasts secrete macrophage colony stimulating factor (M-CSF) and RANKL, which promote the proliferation and survival of osteoclast precursor cells and drive osteoclast differentiation by activating downstream signaling pathways, including MAPK and NF-κB signaling pathways. Furthermore, as osteoclasts dissolve and absorb, key transcription factors such as TGF-β and IGF-1 are released as well. At this stage, medium and low concentrations of TGF-β enhance the expression of key transcription factors, including Runx2 and Osterix, via the TGF-β/Smad2/3 signaling pathway. Furthermore, TGF-β promotes angiogenesis and recruits BMSCs to the injury site. Together, these coordinated events robustly drive osteogenesis and functional bone-vascular coupling.^93^

#### The influence on endothelial cells and bone-vascular coupling

The physiological weakly acidic microenvironment, as a mild stressor, can induce angiogenesis and initiate bone-vascular coupling. Low pH can increase acidic proteases such as CTSK and convert proMMP-9 into catalytic MMP-9.^94^ MMP-9-mediated ECM degradation enhances the release and bioavailability of VEGF, which promotes endothelial cell sprouting and vascular network formation.^95^ VEGF, bFGF, and others can also promote the release of MMP by endothelial cells.

### Pathological acidic microenvironment (pH < 5.5)

If a fracture undergoes impaired local healing or if an implanted polyurethane urea (P-PUU) scaffold degrades too rapidly, excessive acidic by-products are released. Consequently, the pH of the bone microenvironment drops sharply, resulting in pathological acidosis (pH < 5.5).^96^

#### The effects on osteoblasts and osteoclasts

In microenvironmental acidosis, many protons activate the acid-sensing channels (ASICs) and G protein-coupled receptors (OGR1, TRPV) for Ca^2+^ to promote the nuclear translocation of NFATc1, activate the PKC and ERK pathways, promote osteoclast differentiation, and prolong survival time.^97^^,^^98^ In addition, both the ERK and PKC pathways are major nodes in the inflammatory response and can activate classical inflammatory signaling pathways such as NF-κB, release many proinflammatory factors, and further accelerate bone resorption.^99^^,^^100^

Under physiological conditions, osteoclasts continuously pump H^+^ into the bone resorption area using the proton pump V-ATPase to dissolve bone matrix. This process requires a large amount of ATP. When acidosis occurs in the microenvironment, V-ATPase energy increases and osteoclasts differentiate more mitochondria, releasing a large amount of ROS into the microenvironment.^17^^,^^101^ Under low pH and high ROS concentration, osteogenic activity is inhibited, and Runx2 expression increases, while Osterix expression decreases. Runx2 plays a critical role in initiating the differentiation process of MSC into osteoblasts, while Osterix is primarily involved in promoting osteoblast maturation and matrix mineralization during the late phase of osteogenic differentiation.[108] An increase in Runx2 expression combined with a decrease in Osterix expression may impede osteoblast maturation, leading to their retention in an immature state. Furthermore, a study indicated that short-term extracellular acidosis could enhance the stem cell phenotype, thereby promoting the maintenance of BMSCs in a quiescent state and preserving their stem cell characteristics.^102^

#### The influence on endothelial cells and bone-vascular coupling

Persistent acidosis markedly induces apoptosis in bone marrow-derived endothelial progenitor cells (BM-EPCs) while concurrently attenuating the proliferative capacity of the surviving EPC pool. Beyond this precipitous decline in cell numbers, the remaining EPCs also exhibit impaired targeted migration toward injured sites, thereby directly compromising angiogenesis.^103^ Type H vessels, which are pivotal mediators of bone-vascular coupling, rely critically on microenvironmental homeostasis. Both acidosis and the excessive accumulation of ROS inflict direct injury on endothelial cells. Furthermore, they incapacitate the paracrine signaling axis within the bone niche through profound protein denaturation. For example, this dual stress severely suppresses the crucial secretion of PDGF-BB from osteoprogenitor cells.^38^

### Physiological alkaline microenvironment (pH 7.4–8.0)

As the capillary network establishes itself, local metabolic byproducts are efficiently removed, and the osseous microenvironment during the reparative phase gradually assumes a mildly alkaline character (pH 7.4–8.0). This shift promotes osteoblastic differentiation and the oriented deposition of HA crystals.

A mildly alkaline extracellular milieu supports bone formation through several mechanisms. First, it stimulates osteoblast proliferation. When the mouse Pre-OB line MC3T3-E1 was maintained in HEPES-buffered media across a pH gradient from 7.2 to 9.0, cell counts showed a clear positive correlation with rising pH, reaching a maximum at pH 8.4.^104^ Second, alkaline conditions potentiate the activity of ALP, the key enzyme in mineralization. By cleaving inorganic pyrophosphate (PPi) and raising the local concentration of inorganic phosphate (Pi), ALP drives HA precipitation. Arnett and colleagues reported that ALP activity peaks at pH 8.0, coinciding with the greatest accumulation of HA.^105^^,^^106^ Concurrently, osteoclastic resorption is markedly suppressed to preserve the newly formed bone. The mildly alkaline environment neutralizes excess H^+^, thereby inactivating acid-dependent structures and proteases—among them V-ATPase, OGR1, and CTSK. A stable microcirculation not only supplies sufficient nutrients to sustain the high metabolic demands of osteogenesis, but also maintains a healthy bone-vascular coupling structure under a physiologically alkaline environment, marking the transition of fracture healing into the phase of orderly mineralization.

### Pathological alkaline microenvironment (pH > 8.0)

Marked alkalosis during bone defect repair is uncommon and typically iatrogenic in origin. It may arise from overly rapid corrosion of implanted magnesium alloys or from bioactive glass constructs that lack sufficient buffering capacity. In either scenario, a large release of exogenous hydroxyl ions overwhelms the local homeostatic mechanisms of the body, producing a state of regional pathological alkalosis.

Unlike the physiological alkaline range, severe local alkalosis exerts catastrophic effects on osteogenic effector cells, osteoclasts, and the nascent vasculature. Extremely high pH values trigger widespread apoptosis among BMSCs and other osteoprogenitors, thereby disrupting the entire regenerative sequence. Furthermore, the ensuing loss of cellular regulation permits inorganic salts to precipitate in a disordered fashion, yielding inert calcified masses that bear no structural or functional resemblance to biologically deposited HA.^107^

## Crosstalk of various factors: A review from the perspective of bone remodeling

### Acute phase

Accompanying hematoma formation, the local microenvironment rapidly plunges into severe ischemia and hypoxia, triggering a robust inflammatory response. At this stage, high levels of ROS function not merely as metabolic byproducts but as pivotal signaling mediators and effector molecules in bone immunity; they activate ROS-sensitive transcription factors, thereby triggering the NF-κB pathway and driving the cascaded release of pro-inflammatory cytokines such as IL-6 and TNF-α. Notably, the stabilization of HIF-1α under hypoxic stress directs a profound metabolic shift toward glycolysis, significantly upregulating the production of lactic acid. This metabolic byproduct, coupled with the accumulation of local protons, causes localized acidosis. This acidic shift further exacerbates the hostile microenvironment and simultaneously acts as a specific signal for immune cell polarization.

**Figure 5:**
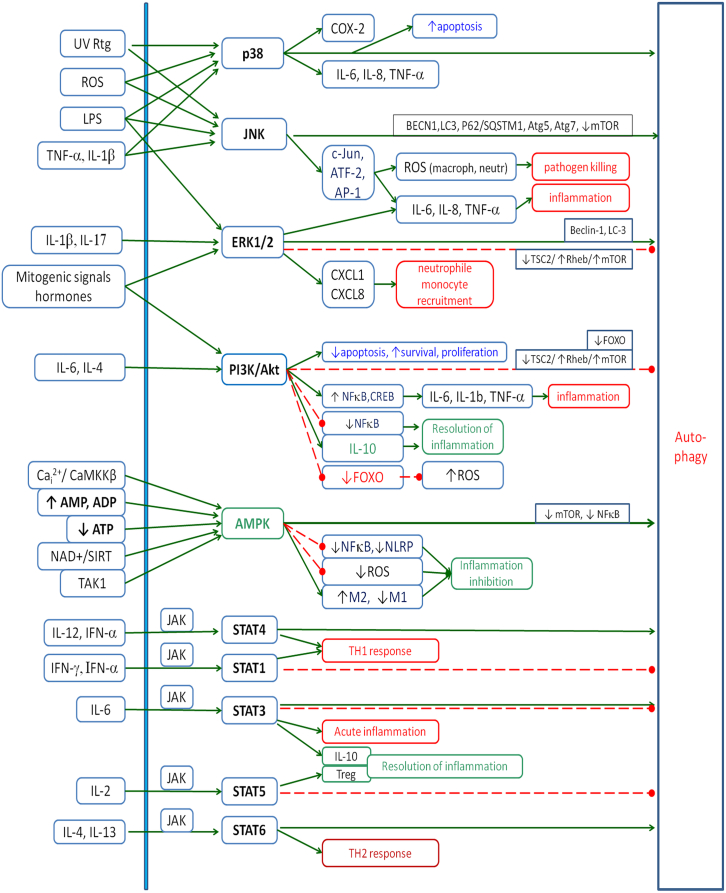
Autophagy signaling pathways activated by diverse stimuli Adapted from Michalak and Michalak.^108^ licensed under the CC BY 4.0 license. Abbreviations: ADP, adenosine diphosphate; AMP, adenosine monophosphate; AMPK, AMP-activated protein kinase; AP-1, activator protein 1; ATF-2, activating transcription factor 2; Atg, autophagy-related; ATP, adenosine triphosphate; BECN1, beclin 1; CaMKKβ, calcium/calmodulin-dependent protein kinase kinase β; COX-2, cyclooxygenase-2; CREB, cAMP response element-binding protein; CXCL, C-X-C motif chemokine ligand; ERK, extracellular signal-regulated kinase; FOXO, forkhead box O; IFN, interferon; IL, interleukin; JAK, Janus kinase; JNK, c-Jun N-terminal kinase; LC3, microtubule-associated protein 1A/1B-light chain 3; LPS, lipopolysaccharide; mTOR, mammalian target of rapamycin; NAD^+^, nicotinamide adenine dinucleotide; NF-κB, nuclear factor kappa-B; NLRP, NLR family pyrin domain containing; PI3K/Akt, phosphoinositide 3-kinase/protein kinase B; Rheb, Ras homolog enriched in brain; ROS, reactive oxygen species; SIRT, sirtuin; SQSTM1, sequestosome 1; STAT, signal transducer and activator of transcription; TAK1, transforming growth factor-beta-activated kinase 1; TH, T helper; TNF, tumor necrosis factor; Treg, regulatory T cell; TSC2, tuberous sclerosis complex 2; UV, ultraviolet.

**Figure 6:**
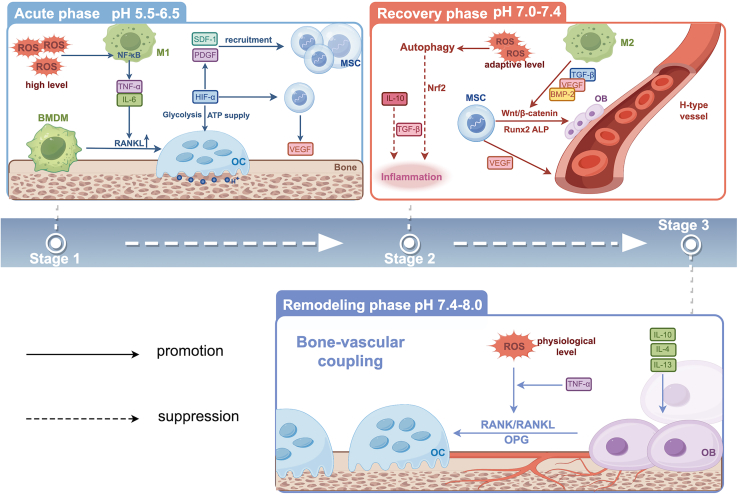
Integrated spatiotemporal landscape of bone regeneration under dynamic microenvironments (drawn by figdraw) Abbreviations: ROS, reactive oxygen species; MSCs, mesenchymal stem cells; BMDM, bone marrow-derived macrophage; OC, osteoclast. Stage 1 (acute phase, pH 5.5–6.5): Elevated ROS levels promote M1 macrophage polarization, in which inflammatory cytokine signaling drives osteoclastic bone resorption and MSC recruitment.Stage 2 (repair phase, pH 7.0–7.4): Adaptive ROS levels exert anti-inflammatory effects through autophagy. M2 macrophages, together with multiple cytokines, synergistically promote H-type angiogenesis and osteogenic differentiation. Stage 3 (remodeling phase, pH 7.4–8.0): Physiological ROS levels mediate bone-vascular coupling and establish bone repair homeostasis through regulation of the RANK/RANKL/OPG axis.

During this process, autophagy plays a dual role: While it initially enables cells to resist various stress-inducing stimuli, the induction of excessive autophagy under extreme oxidative stress can impair cell survival and exacerbate early tissue damage. (Figure 5) This extreme microenvironment is characterized by localized acidosis, excessive ROS, and high concentrations of TNF-α and IL-6. It modulates the proliferation and differentiation of osteoclasts through distinct mechanisms, providing a fundamental basis for their activation. Simultaneously, ROS activate the IKK complex under oxidative stress in a concentration-dependent manner, synergizing with inflammatory factors to upregulate RANKL expression and strongly promoting the differentiation of osteoclast progenitors for efficient debridement. Centrally, the HIF-1α-dependent release of SDF-1 and PDGF establishes a spatial chemotactic gradient essential for the homing of distant MSCs and EPCs. Furthermore, the interplay between autophagy and VEGF secretion optimizes the local microenvironment, facilitating robust vascular sprouting and coordinating the early stages of the regenerative cascade (Figure 6).

### Recovery phase

As the microenvironment transitions from a pro-inflammatory to an anti-inflammatory state, the focus of bone repair shifts toward reconstruction. This transition is marked by a significant dampening of pro-inflammatory signals and the emergence of anti-inflammatory cytokines, such as IL-10 and TGF-β, which actively promote tissue resolution. During this phase, the attenuation of local ROS to an adaptive level rehabilitates favorable autophagy, which enables cells to resist various stress-inducing stimuli. Mitophagy mitigates oxidative damage by selectively clearing damaged mitochondria, thereby priming the Nrf2 signaling pathway. Crucially, Nrf2 activation further inhibits ferroptosis by upregulating the glutathione peroxidase 4 (GPX4) pathway, thereby safeguarding osteoblast viability against lipid peroxidation-induced membrane damage.^109^ In this homeostatic environment, functional VEGFR2 signaling responds to VEGF gradients from MSCs, driving the extension of H-type vessels. This process is further amplified by the synergy between autophagy and VEGF, which exerts robust pro-angiogenic effects. Simultaneously, MSCs differentiate into mature osteoblasts, where autophagy must be maintained at an appropriate level to ensure proteostasis and osteoid secretion. This bidirectional crosstalk between OBs and endothelial cells achieves the spatiotemporal coupling of angiogenesis and osteogenesis, shifting bone repair from a survival phase to a reconstructive phase (Figure 6).

### Remodeling phase

In the final remodeling phase, the bone microenvironment transitions from functional reconstruction to long-term homeostasis. At this stage, physiological levels of ROS function as both signaling and effector molecules, synergizing with baseline pro-inflammatory cytokines, specifically TNF-α and IL-1β, to precisely regulate the RANKL/OPG axis. This controlled signaling maintains appropriate osteoclast activity, ensuring the remodeling of woven bone into mature lamellar bone. Meanwhile, autophagy in osteocytes must be maintained at an appropriate level to ensure their survival and functional stability under microenvironmental stressors within the mineralized matrix. Consequently, the integration of ROS signaling, inflammatory baselines, and autophagic regulation achieves the transition of bone repair from structural reconstruction to homeostatic maintenance.

In summary, the phenomena of bone-vascular uncoupling can be elucidated through two distinct dimensions of mismatch. First, the age-related reversal of HIF-1α efficacy is fundamentally explained by temporal uncoupling. In the aged microenvironment, excessive ROS accumulation disrupts the synchronized metabolic transition. This disruption ultimately leads to a breakdown in the timing of signaling crosstalk.^32^ Second, anatomical heterogeneity is explained by spatial uncoupling inherent to the ossification pathway. In the endochondral ossification of long bones, the cartilaginous template dictates a mandatory spatial coupling. Conversely, the intramembranous ossification of flat bones allows for the independent and non-synchronized progression of angiogenesis and osteogenesis. Acknowledging these temporal and spatial nuances is essential for a comprehensive understanding of the endogenous self-repair mechanism (Figure 6).^5^

## Obstacles to clinical translation and countermeasures

A major challenge arises from model heterogeneity. Small animal models, primarily rodents such as mice and rats, are widely used in basic research due to their low cost, ease of maintenance, and well-defined genetic backgrounds. Among these, the rat calvarial defect model is a classic non-load-bearing critical-size defect model. Owing to its relatively limited vascular supply and absence of mechanical loading, it is particularly suitable for evaluating the intrinsic osteoinductive and angiogenic potential of various scaffold systems. However, rodents lack a Haversian system and therefore only exhibit simple bone formation, failing to recapitulate the cortical bone remodeling that is critical in human bone repair. In addition, rodents possess a much stronger intrinsic healing capacity than humans, which increases the risk of false-positive outcomes. Therefore, large animal models (e.g., canine and porcine) remain the “gold standard” for translational bone tissue engineering, despite their longer experimental periods and higher costs. Unlike rodents, these models possess a functional Haversian system, and their bone turnover rates and biomechanical loading conditions closely resemble those in humans.^110^

In addition to the limitations of animal models, differences in ossification pathways also represent a major barrier to clinical translation. Current bone biology research largely focuses on inducing MSCs to directly differentiate into osteoblasts and secrete bone matrix, which resembles intramembranous ossification observed in craniofacial bones.^111^ However, most clinical bone defects occur in long bones, for which such intramembranous ossification-based strategies are not suitable. Long bone repair predominantly proceeds via endochondral ossification, in which MSCs first differentiate into chondrocytes, produce a cartilaginous matrix, and subsequently undergo mineralization. During this process, chondrocytes become hypertrophic, exhibiting enhanced osteoinductive and pro-angiogenic capacities, particularly in promoting the formation of type H vessels. This ossification mode is therefore superior in repairing large bone defects. In contrast, current bone tissue engineering strategies often fail due to insufficient vascular ingrowth into the defect core, leading to central necrosis.^112^

Furthermore, the lack of quantifiable criteria for evaluating the restoration of “bone-vascular coupling” remains a critical limitation in clinical translation. In basic and animal studies, techniques such as micro-CT angiography and immunofluorescence staining of bone tissue are commonly used to quantify the volume of type H vessels and their spatial relationship with osteoprogenitor cells at the microscopic level.^113^ However, these invasive methods are generally not feasible in human clinical trials. Clinically, the assessment of bone defect healing still relies heavily on conventional non-invasive imaging techniques, such as X-ray and computed tomography (CT), which can only detect late-stage macroscopic changes, including cortical bridging and alterations in bone density. Early microscopic events, such as microvascular infiltration and cellular activity, cannot be effectively visualized. In addition, routine serum biomarkers (e.g., ALP) mainly reflect systemic bone metabolism and lack the specificity to indicate the local bone-vascular coupling status within the defect region.

With aging, the bone marrow microenvironment undergoes irreversible degenerative changes, including a reduction in the number of type H vessels and decreased secretion of key bone-vascular coupling factors such as PDGF-BB, leading to a marked decline in bone regenerative capacity.^33^ Moreover, underlying diseases also significantly impair bone healing. For example, chronic hyperglycemia in diabetes results in microcirculatory dysfunction, creating a hypoxic microenvironment and inducing excessive ROS production. This, in turn, promotes osteoblast apoptosis and stimulates osteoclastogenesis, ultimately impairing bone healing.^114^

Although systemic pharmacological interventions for managing bone loss have become a clinical consensus, exogenous delivery of growth factors such as VEGF and PDGF-BB has been shown to counteract the negative effects of aging and comorbidities on osteogenesis.^115^ However, a major challenge lies in confining their activity to the defect site while achieving release kinetics that align with the physiological timeline of bone formation. To address this issue, advanced delivery strategies have been proposed. Core-shell microspheres can physically separate different therapeutic agents, enabling spatiotemporally controlled delivery. For hypoxic conditions, bone microenvironment-regulating hydrogels,^116^ such as the CPP-L/GelMA system, can provide sustained oxygen release while simultaneously scavenging ROS and buffering pathological pH imbalances. Both *in vitro* and *in vivo* studies have demonstrated that such hierarchical delivery systems can maintain stable oxygen release for over two weeks, effectively avoiding the initial burst release phenomenon. This strategy may also serve as a reference for the controlled delivery of growth factors such as VEGF in clinical applications.^116^

In summary, to bridge the gap between reductive *in vitro* cultures and disparate animal models, bone-on-a-chip (BOC) technology has been progressively developed. Compared with traditional models, BOC systems can incorporate patient-derived cells and simulate physiological mechanical stimuli, thereby improving experimental relevance. By integrating additively manufactured structural parameters (e.g., microporosity and topological geometry), these platforms can better recapitulate the complex *in vivo* fluidic environment, enabling more accurate assessment of how physical microenvironments regulate bone ingrowth and angiogenesis.^117^ Furthermore, integration with artificial intelligence (AI) allows high-throughput image analysis, extracting micron-scale parameters such as cell distribution, matrix mineralization, and vascular architecture for evaluating therapeutic efficacy. However, due to the simplified experimental environment and the current lack of extensive validation, BOC technology cannot yet fully replace animal models.

## Conclusion and future perspectives

Healing critical-sized bone defects is a highly orchestrated and timed process of bone remodeling, whose main mechanism is the equilibrium between osteoclastic resorption and osteogenesis based on bone-vascular coupling. We have described the regulatory effect of different pathologic bone microenvironments on this equilibrium. In the early postfracture phase, hypoxia, pro-inflammatory signaling, localized oxidative stress, and acidic conditions facilitate clearing the necrotic tissue. In the late and early stages of repair, release of various growth factors and bone-vascular coupling occur, corresponding to an upregulation of antioxidant defenses and autophagic flux. Furthermore, the pH of the bone microenvironment gradually increases, and conditions allow the accumulation of HA in order. Although this review provides a qualitative characterization of the temporal dynamics of these regulatory factors and highlights how concentration-dependent mechanisms often account for their context-dependent biphasic effects reported in various studies, significant translational challenges remain. Specifically, current methodologies still struggle to precisely quantify *in vivo* parameters and implement targeted, high-resolution therapeutic modulation. This limitation arises from the inherent heterogeneity of animal models, the anatomical specificity of defect sites, and the intricate spatiotemporal signaling crosstalk within the microenvironment. Taken together, a more profound interrogation of the regulatory networks governing the bone microenvironment, when coupled judiciously with emerging technological platforms, is poised to surmount extant hurdles in clinical translation and to chart novel avenues for the therapeutic management of critical-size bone defects.

## Acknowledgments

We sincerely thank Prof. Changyong Yuan from the Affiliated Stomatological Hospital of Xuzhou Medical University for his valuable guidance and critical review during the revision of this manuscript. We also extend our gratitude to the editor and the anonymous reviewers for their insightful and constructive comments, which greatly enhanced the quality of this paper.

This work was supported and funded by the Jiangsu Provincial Health Research Project (Z2023015) and the general project of Basic Research Plan in Xuzhou City (KC23069).

## Author contributions

Conceptualization, Z.X. and M.Z.; methodology, Z.X., and M.Z., J.L., and R.D.; formal analysis, Z.X.; investigation, M.Z. and J.L.; resources, R.D.; data curation, Z.X.; writing – original draft preparation, Z.X. and M.Z.; writing—review and editing, Z.X., M.Z., and J.L.; visualization, M.Z. and J.L.; supervision, R.D. and J.W.; project administration, Z.X.; funding acquisition, R.D. All authors have read and agreed to the published version of the manuscript.

## Declaration of interests

The authors declare that they have no financial interests.
